# The Integration of Sex and Gender Considerations Into Biomedical Research: Lessons From International Funding Agencies

**DOI:** 10.1210/clinem/dgab434

**Published:** 2021-06-17

**Authors:** Jamie White, Cara Tannenbaum, Ineke Klinge, Londa Schiebinger, Janine Clayton

**Affiliations:** 1 Office of Research on Women’s Health, National Institutes of Health, Bethesda, Maryland 20817, USA; 2 Institute of Gender and Health, Canadian Institutes of Health Research, Montreal, Quebec H3W 1W5, Canada; 3 Rapporteur H2020 Gendered Innovations 2, European Commission, Brussels, Belgium; 4 History of Science, Stanford University, Stanford, California 94305, USA

**Keywords:** policy, sex, and gender, based analysis, SABV, research funding agencies

## Abstract

To improve the outcomes of research and medicine, government-based international research funding agencies have implemented various types of policies and mechanisms with respect to sex as a biological variable and gender as a sociocultural factor. After the 1990s, the US National Institutes of Health (NIH), the Canadian Institutes of Health Research (CIHR), and the European Commission (EC) began requesting that applicants address sex and gender considerations in grant proposals, and offering resources to help the scientific community integrate sex and gender into biomedical research. Although it is too early to analyze data on the success of *all* of the policies and mechanisms implemented, here we review the use both of carrots (incentives) and sticks (requirements) developed to motivate researchers and the entire scientific research enterprise to consider sex and gender influences on health and in science. The NIH focused on sex as a biological variable (SABV) aligned with an initiative to enhance reproducibility through rigor and transparency; CIHR instituted a sex- and gender-based analysis (SGBA) policy; and the EC required the integration of the “gender dimension,” which incorporates sex, gender, and intersectional analysis into research and innovation. Other global efforts are briefly summarized. Although we are still learning what works, we share lessons learned to improve the integration of sex and gender considerations into research. In conjunction with refining and expanding the policies of funding agencies and mechanisms, private funders/philanthropic groups, editors of peer-reviewed journals, academic institutions, professional organizations, ethics boards, health care systems, and industry also need to make concerted efforts to integrate sex and gender into research, and we all must bridge across silos to promote systemwide solutions throughout the biomedical enterprise. For example, policies that encourage researchers to disaggregate data by sex and gender, the development of tools to better measure gender effects, or policies similar to SABV and/or SGBA adopted by private funders would accelerate progress. Uptake, accountability for, and a critical appraisal of sex and gender throughout the biomedical enterprise will be crucial to achieving the goal of relevant, reproducible, replicable, and responsible science that will lead to better evidence-based, personalized care for all, but especially for women.

In the past 40 years, paradigms in health research have shifted from studying mechanisms and treatments in predominantly male participants to investigating complex systems in heterogeneous populations. Concurrently, research funding agencies have questioned the overreliance on male patients, animals, and cells; the lack of transparency and reproducibility of studies; inattention to sex effects; and inconsistent reporting of sex-specific findings, all of which have compromised the rigor, reproducibility, and generalizability of basic science and clinical studies. In addition, the notions that females are just smaller males and that their hormonal levels interfere with the interpretation of data because of greater variability due to sex have been disproven (1). When results from males are improperly generalized to females, the missing results for females and inattention to sex and gender lead to errors and false conclusions. Sex differences in physiology, pharmacology, and disease progression form the basis of understanding and developing appropriate treatments for many endocrine disorders and conditions (2). Studies with irreproducible results, biases, or unforeseen toxicities put people at risk of being harmed, contributing to the erosion of public trust in medicine and science (3).

A hallmark of 21st century biomedical research has been the integration of sex- and gender-based analysis (SGBA) into study design (4). And now with the globalization of science, it is crucial that researchers consider, collect, characterize, and communicate sex and gender influences on health within biomedical research. The current pandemic has showcased the importance of SGBA to health outcomes. Presently, evidence shows that factors related both to sex and gender, along with sex and gender interactions, play into the risk of mortality and morbidity of COVID-19 patients (5-7). Results also show the interplay of endocrinological and biological factors, such as sex hormone levels and immune responses, as well as societal factors, on diseases (8, 9). Factors related to sex, age, and chronic metabolic disease can produce a deadly confluence of dysregulated immunometabolism and inflammation in patients who have contracted COVID-19 (10). The disaggregation of data relevant to those factors is imperative to understand how they influence health outcomes and the underlying causes and effects of disease.

It is important to note that, although the terms *sex* and *gender* are often used interchangeably, “sex” refers to biological attributes, such as chromosomes, gonadal organs, and endogenous hormonal profiles that distinguish organisms as male, female, intersex, and hermaphrodite, whereas “gender” refers to socially and culturally constructed factors that shape behaviors, stereotypes, and attitudes across societies over time (11). Sex, gender, and their interactions influence health.

Several government-based research funding agencies developed policies that integrate sex as a biological variable (SABV) and gender as a sociocultural determinant of health into medical research. We review the historical development of research-related policies of countries around the world, with a focus on the United States, Canada, and the European Union (EU) (Table 1).

**Table 1.** Comparative historical overview of key events and policy implementation

Epoch	United States	Canada	European Union
1990s	1990: ORWH formed	1995: Commitment to gender-based analysis plus on all legislation, policies, and programs	1997: Treaty of Amsterdam, which reported on the status of women across EU countries in terms of equal pay, maternity leave, education, and other issues
	1993: NIH Revitalization Act mandated inclusion of women and minorities in clinical research		1998: Sector on women and science created to mainstream gender throughout the EC
	1994: NIH Guidelines on the Inclusion of Women and Minorities as Subjects in Clinical Research: “NIH Inclusion Policy” developed NIH Guide, Vol. 23, No. 11, March 18, 1994		1999: Communication: Women in Science, Mobilising Women to Enrich European Research
			1999: European Technology Assessment Network working group on Women and Science delivered its report: Science Policies in the EU: Promoting Excellence through Mainstreaming Gender Equality
2000s	2001: NIH and FDA fund the IOM report, “Exploring the Biological Contributions to Human Health: Does Sex Matter?” (12)	2000: CIHR is formed; 13 funding institutes are designated by an act of parliament with equal budget and decision-making capacity, one of which is the CIHR IGH. A GBA policy that applies broadly to the entire government of Canada is implemented.	2000: Gender Impact Assessment Studies for FP5
	NIH Inclusion Policy amended Oct. 9 (2001 NOT-OD-02-001). The amended policy provides additional guidance on the analyses and reporting of analyses of sex/gender, racial/ethnic, and other effects for NIH-defined phase 3 clinical trials.	2006: CIHR begins to integrate SGBA into applications through funding policy and applicant and reviewer guidelines.	2001: First Gender in Research Conference
	2006: Organization for the Study of Sex Differences formed	2009: The Government of Canada’s Health Portfolio updates its policy to an SGBA policy to differentiate sex as a biological variable from gender as a sociocultural determinant of health.	2002-2006: FP6 requirement to address the “gender dimension,” which integrates both sex and gender analysis into research design, where appropriate (later referred to as “sex and/or gender analysis”). This requirement forms the third objective of the EC’s Gender Equality Policy.
	2006: NIH ORWH and FDA OWH deploy an online continuing education course: “Science of Sex and Gender in Human Health”		2004-2008: Gender Monitoring Studies
	2007: NIH announces SCOR on Sex Differences to integrate basic, preclinical, clinical, and translational research to facilitate innovative, interdisciplinary studies on sex differences and on diseases and conditions that affect women’s health.		2009: Treaty of Lisbon: All policy areas must ensure equality of women and men.
2010s	2010: Moving into the Future with New Dimensions and Strategies for Women’s Health Research—A Vision for Women’s Health Research (NIH strategic plan for women’s health and sex/gender difference research); NIH supports IOM’s Sex-Specific Results Reporting Workshop and Report	2010: Reporting on sex/gender in study design during the submissions of CIHR grant applications is implemented.	2011: The expert group, Innovation through Gender, is formed.
	2014: NIH ORWH and FDA OWH update and expand the online course (currently being updated again)	2014: Sex and Gender Champion program launched	2013: GENDER-NET is formed to promote gender equality through structural change in research institutions and to integrate sex and gender analysis into research. The network consists of 16 organizations from 12 European countries and Canada.
	2013: NIH Sex/Gender Administrative Supplement funding began, to encourage sex/gender comparisons in preclinical and clinical studies already being conducted.	2015 and 2017: Catalyst grants launched	2014-2020: FP8 (Horizon 2020); AGG launched
	2015: Rigor and Reproducibility policy (13) requiring applicants to report plans to balance male and female cells and animals in preclinical studies instituted (14)	2016-2017: New SGBA Research Action Plan to better integrate SGBA in CIHR’s research funding programs is developed and implementation begins.	2017: GENDERACTION project launched; GENDER-NET Plus reformed
	2016: Consideration of SABV in NIH-funded Research policy, which applies to vertebrate and human studies, is instituted (15); 21st Century Cures Act passed (16)	2016: Completion of sex and gender training modules as an eligibility criterion for specific funding opportunities	2018: Proposal for Horizon Europe (FP9) 2021 to 2027 published.
	2017: Amendment to NIH Policy and Guidelines on the Inclusion of Women and Minorities as Subjects in Clinical Research now requires recipients conducting applicable NIH-defined phase 3 clinical trials to ensure results of valid analyses by sex/gender, race, and/or ethnicity are submitted to ClinicalTrials.gov (16).	2018: SGBA research action plan, includes peer review assessment of the appropriateness of sex and gender integration in investigator-initiated project grants through structural changes to the peer-review assessment form and capacity building among reviewers.	2018: The Expert Group to Support Sex, Gender, and Intersectional Analysis in Horizon Europe is formed.
	2018: Inclusion Across the Lifespan policy (17); SCOR is converted to SCORE, which requires a Career Enhancement Core to support training of innovative sex-based and informed translational research methods and best practices and to provide leadership in the development and promotion of standards and polices for the consideration of sex differences.	2018: CIHR makes systematic monitoring of the integration of sex and gender considerations into CIHR-funded research a performance indicator in its departmental results accountability framework to government.	
	2019: Advancing Science for the Health of Women: 2019 to 2023 trans-NIH Strategic Plan for Women’s Health Research was released; ORWH issues first Funding Opportunity Announcement for R01 (RFA-OD-19-029) that solicits applications on the influence and intersection of sex and gender in health and disease.	2019: CIHR approves IGH’s sex and gender science strategy, which includes an interdisciplinary cadre of Sex and Gender Science Chairs.	
2020s		2020: CIHR introduces new gender policies to mitigate the effects of the COVID-19 pandemic (18).	2020: The first Horizon Europe Strategic Plan (2021-2024) includes 2 new developments: 1) sex and gender analysis in research design is required in all applications unless its nonrelevance is justified; and 2) institutions must implement a GEP to be eligible to apply for EC funding. The GEP includes sex, gender, and intersectional analysis in research design (20).
		2021: CIHR launches its 2021 to 2031 Strategic Plan that emphasizes equity and attention to sex and gender as an integral component of scientific excellence (19).	

Abbreviations: AGG, Advisory Group for Gender; CIHR, Canadian Institute for Health Research; EC, European Commission; EU, European Union; FDA, US Food and Drug Administration; FP, Framework Programme; GBA, gender-based analysis; GEP, Gender Equality Plan; IGH, Institute of Gender Health; IOM, Institute of Medicine; NIH, National Institutes of Health; ORWH, Office of Research on Women’s Health; OWH, Office of Women’s Health; SABV, sex as a biological variable; SCOR, Specialized Centers of Research; SCORE, Specialized Centers of Research Excellence; SGBA, sex- and gender-based analysis.

**Table 2.** Comparison of policies, mechanisms, and lessons learned

	United States	Canada	European Union
Policy	Consideration of SABV in NIH-funded research (15)	Sex- and gender-based analysis (21)	Gender dimension (sex and/or gender analysis)
Definition	Researchers are expected to perform the following:	Sex and/or gender considerations are integrated into every step of the research project, including project rationale, experimental design, methods, analysis, and knowledge translation and dissemination.	Researchers are required to integrate intersectional sex and gender analysis into each stage of the research and innovation process, where relevant.
	1) consider the influence of sex and/or gender when formulating research questions		
	2) review the literature for the influence of sex and/or gender		
	3) account for the influence of sex and/or gender in study design		
	4) incorporate males and females into studies or justify using only one sex and/or gender		
	5) collect, analyze data, and report data disaggregated by sex and/or gender		
	6) characterize the influence of sex and/or gender in the interpretation of results		
	7) communicate appropriately generalized findings.		
Requirement	Required for vertebrate animal and human studies supported by NIH	Included in instructions to applicants. Questions on the application form for all competitions ask whether sex and/or gender are accounted for in the proposal. Justification is required for the inclusion or omission of sex and/or gender.	Horizon Europe requires all proposals to include intersectional sex and gender analysis, unless the exclusion is justified. Institutions must implement a GEP for their PIs to be eligible to apply for EC funding.
	Strong justification required for single-sex studies		
Funding opportunities	1) Administrative supplements enable researchers to add sex/gender analyses to ongoing research.	1) Investigator-initiated grants that seek ideas with the greatest potential to advance health-related fundamental or applied knowledge, health research, health care, health systems, and/or population health and policy research require consideration of sex and/or gender.	1) Supplements for training in gender analysis.
	2) SCOR(E) program integrates basic, preclinical, clinical, and translational research and serves as a vital hub for education and dissemination of innovative sex-based and informed translational research methods and best practices.	2) Strategic team grants on personalized health, immunology, microbiome, developmental origins of chronic disease, healthy cities, and others	2) The EC has funded many projects to support and disseminate the gender dimension and the GEPs, including Gender Academy Training (22) and those discussed in the text.
	3) U3 supplements support active NIH parent grants for 1 year to address research on the effect of sex/gender influences at the intersection of several social determinants, including race/ethnicity, socioeconomic status, education, health literacy, and other social determinants in human health and illness.	3) Strategic catalyst grants for basic scientists are intended to stimulate research on a particular topic.	
	4) Common Fund Administrative Supplements	4) Sex and Gender Science Chair grants are intended to form a cadre of experts across all disciplines	
	5) Investigator-initiated funding opportunities such as The Intersection of Sex and Gender Influences on Health and Disease (R01 Clinical Trial Optional)–RFA-OD-19-029		
Mechanisms	1) Required in all relevant applications within the research strategy section and assessed as part of the application’s score	1) For specific competitions, the nominated principal applicant is required to complete an online training module on sex and gender integration.	1) The Funding and Tenders portal contains all relevant information for applicants and evaluators, with links to appropriate tools.
	2) Training of investigators and reviewers via online courses	2) For specific competitions, a sex and gender champion is required on the research team.	2) Including the gender dimension is the default requirement for Horizon Europe.
	3) Governance structures	3) Requirement for a cross-cutting sex and gender platform across specific strategic consortium grants	3) Training in integrating the gender dimension has been given to commission staff (program writers, members of executive agencies).
	4) Specific language is embedded in relevant funding opportunity announcements	4) Embedded within the objectives and evaluation criteria for specific strategic funding opportunities	4) Training in integrating the gender dimension has been organized for national contact points in member states.
	5) Trans-NIH Strategic Plan for Advancing the Health of Women		5) EC monitors the percentage of projects with a gender dimension in the project design.
Peer-review processes	1) Methods and study design of the application are reviewed for compliance with SABV; overall score for approach reflects how issues are addressed.	1) Chairs and scientific officers on project peer-review committees are required to complete the sex and gender online training modules.	1) Evaluator briefings
	2) Decision tree guidelines provided to assist peer review 3) Summary statement reflects study section discussion and reviewer critiques	2) A designated sex and gender expert is assigned to each project peer-review committee.	2) Training modules on avoiding implicit bias in evaluations
		3) Structural change on peer-review forms requires peer reviewers to comment on the strength or weakness of integration of sex and gender and to provide recommendations for improvement.	
		4) Included in the evaluation criteria for specific strategic funding opportunities	
		5) Members of each peer-review committee receive discipline-specific training on how to critically appraise sex and gender in research content	
Lessons learned	1) Training needed to understand why sex and gender considerations are important; training and resources need to help researchers and reviewers understand the SABV policy and to promote adherence.	1) Sex- and gender-specific funding policies provide the most leverage for funders to influence applicant and evaluator behavior	1) The gender dimension in research design is easily ignored unless required. The new Horizon Europe requires this element in proposals, unless a justification is provided.
	2) Importance of preparing the scientific community for a shift in science culture	2) There is no magic formula. Serial multicomponent interventions are required to gradually shift culture over time for the research community	2) Researchers, evaluators, and agency staff require training in sex, gender, and intersectional analysis.
	3) Understanding that many sectors and disciplines are at different stages of integrating sex and gender into research approaches; hence, “interventions” need to be tailored or entity based on where a particular field is in the process.	3) Requiring evaluators to provide checkbox (strength, weakness, not applicable) and written feedback to applicants on whether sex and gender were appropriately considered improves accountability. Asking evaluators to discuss this feedback with each other during the peer review committee increases accountability among peer reviewers to provide quality assessments.	3) The agency must fund training resources, such as Gendered Innovations, the Gender Academy, and Gender-NET ERA-NET.
	4) Identifying and enlisting champions in various fields can be an effective strategy.	4) Reviewers and applicants benefit from discipline-specific tools to increase capacity.	4) Evaluation criteria for proposals must be tailored to each research and innovation domain.
	5) Major cultural shifts take time regardless of the structural and process changes that are implemented to facilitate them.	5) Sex and gender champions on research teams and review committees add value; however, in isolation, they are not a feasible solution as they exist in insufficient numbers to populate all team grants and committees.	5) The output of funded research (ie, peer-reviewed articles) should be monitored for quality integration of sex, gender, and intersectional analysis and be reported in the biennial EU She Figures (23).

Abbreviations: EC, European Commission; EU, European Union; FDA, US Food and Drug Administration; GEP, Gender Equality Plan; NIH, National Institutes of Health; PI, principal investigator; SABV, sex as a biological variable; SCOR, Specialized Centers of Research; SCORE, Specialized Centers of Research Excellence; SGBA, sex- and gender-based analysis; U3, Understudied, Underrepresented, and Underreported.

At the US National Institutes of Health, the Office of Research on Women’s Health (ORWH) implemented the 2019 to 2023 Trans-NIH Strategic Plan for Women’s Health Research: Advancing Science for the Health of Women to ensure that sex and gender influences are integrated into biomedical research to enhance research relevant to the health of women; that every woman receives evidence-based care tailored to her own needs and circumstances through the appropriate representation of all women in biomedical research; and that women advance in biomedical careers. At the Canadian Institutes of Health Research (CIHR), the Institute of Gender and Health (IGH) plays a leadership role in advancing knowledge and building capacity in sex and gender science across disciplines and career stages and accelerates the uptake of evidence in practice and policy. At the European Commission (EC), Horizon Europe seeks to integrate sex, gender, and intersectional analysis into research and innovation to improve the scientific quality and societal relevance of science and technology. All agencies strive to incorporate sex and gender considerations into each stage of the research process, from study design through data collection and analysis to reporting, resulting in scientific outputs that are more rigorous, reproducible, inclusive, and applicable (3).

## Policies on Sex and Gender Considerations in Research

### National Institutes of Health Office of Research on Women’s Health

In 1993, the NIH Revitalization Act mandated the inclusion of women and minorities in clinical research (Table 1). In 2015, the NIH introduced an initiative to enhance reproducibility through rigor and transparency by clarifying expectations for experimental designs and by requiring researchers to account for relevant biological variables (such as sex) and to authenticate key biologic agents (13).

In 2016, the US Congress passed the 21st Century Cures Act to accelerate the discovery and delivery of cures (16). The NIH also issued “Consideration of SABV in NIH-funded Research,” a new policy requiring awardees to account for SABV in research design, analysis, and reporting in vertebrate animal and human studies (15). It does not require investigators to look specifically for sex differences but, rather, to rigorously account for sex and justify single sex studies, to enhance the knowledge base and inform subsequent investigations. NIH Policy and Guidelines on the Inclusion of Women and Minorities as Subjects in Clinical Research now require recipients conducting applicable NIH-defined phase 3 clinical trials to ensure that results of valid analyses by sex/gender, race, and/or ethnicity are submitted to ClinicalTrials.gov (24).

### Canadian Institutes of Health Research Institute of Gender and Health

The government of Canada requires all departments to consider how government policies and programs affect people of different genders (25). CIHR is a signatory on the Government of Canada’s Health Portfolio’s SGBA policy, which differentiates the biological factors associated with sex from the sociocultural factors associated with gender (21). SGBA has 2 facets: the first pertains to participation, that is, who gets funding and whether those decisions are made in an equitable way, and the second pertains to research, that is, what or who is studied and will those studies generalize across sexes and genders?

### European Commission Horizon Europe

The EC first attempted to integrate the gender dimension into Framework Programme 6 (2002-2006; see Table 1). The EC’s “gender dimension” is the third objective of its Gender Equality Policy. Whereas CIHR and NIH policies apply only to health and medicine, the EC policy applies across all domains of priority-driven science and technology. In its current program, Horizon Europe (2021-2027), sex and gender analysis applies across its major research clusters: Health; Culture, Creativity, and Inclusive Society; Civil Security for Society; Digital, Industry, and Space; Climate, Energy, and Mobility; and Food, Bioeconomy, Natural Resources, Agriculture, and Environment; and across its major research mission areas: Cancer; Adaptation to Climate Change, including Societal Transformation; Healthy Oceans, Seas, Coastal and Inland Waters; Climate-Neutral and Smart Cities; and Soil Health and Food.

### Other Global Efforts

Several European national funding agencies have implemented policies for SGBA; for a full list of policies, see reference (26). An Australian group recently called for its country’s policies to align with those of North American and European countries (27).

## Integrating Sex/Gender into Research: Incentives and Requirements

### National Institutes of Health Office of Research on Women’s Health

Accounting for SABV begins when developing research questions, and consideration of sex is critical to the interpretation, validation, and generalizability of research findings. The NIH’s SABV policy is incorporated into peer review processes, governance, programming, and oversight (28). Before the policy was developed, the NIH offered supplements to existing funded studies to investigate sex and gender; the additional funds were for animals/subjects of the opposite sex/gender, to increase the sample size, and/or to perform secondary data analyses. Revised grant application instructions and forms and reviewer guidelines provide information on how and where to consider SABV. Assessment of how well SABV is accounted for in the research strategy contributes to an application’s score. NIH institutes and centers also incorporate topic-related SABV language into funding opportunities and strategic planning processes.

The NIH has a longstanding commitment to SABV through funding. In 2003, the NIH began offering $1 million grants to establish Specialized Centers of Research (SCOR) on sex differences (see Tables 1 and 2). In 2013, the ORWH began offering supplements to NIH grantees to catalyze new investigations of sex/gender influences across many fields. Through 2019, ORWH invested more than $37 million, supporting 375 principal investigators from every eligible institute and center. In 2014, the NIH Common Fund provided more than $5 million in similar supplements to foundational disease-agnostic platforms.

The NIH conducted its first SABV workshop in October 2017, featuring NIH investigators who presented their findings garnered from applying SABV perspectives to their research. To facilitate application of the policy in multiple contexts, other institutes and centers conducted SABV workshops, formed area-specific working groups, and published review papers. ORWH convenes and disseminates information to key stakeholders by hosting seminars, workshops, and training sessions and by publishing blog posts, a newsletter, articles, and commentaries. ORWH developed free interactive e-learning courses (see Table 3)—the SABV Primer, on how to consider SABV in all stages of the biomedical research spectrum and Bench to Bedside: Integrating Sex and Gender to Improve Human Health, which helps users understand sex and gender influences on health and diseases. The module on endocrinology presents information on sex and gender differences in 3 conditions—osteoporosis, type 2 diabetes, and differentiated thyroid cancers—and explains the impact of sex differences on the clinical presentation and treatment of endocrine and metabolic disorders. It also details knowledge gaps in endocrine and metabolic research to help develop ideas for future preclinical and clinical research studies. Lastly, in 2019, ORWH issued its first Funding Opportunity Announcement for R01 grants that support disease-agnostic research on the intersection of sex and gender across scientific disciplines.

**Table 3.** Resources

Agency	Type of resource and links
ORWH	Consideration of SABV in NIH-funded research
	https://grants.nih.gov/grants/guide/notice-files/NOT-OD-15-102.html
	Enhancing reproducibility through rigor and transparency
	https://grants.nih.gov/grants/guide/notice-files/NOT-OD-15-103.html
	Implementing rigor and transparency in NIH and AHRQ research grant applications
	https://grants.nih.gov/grants/guide/notice-files/NOT-OD-16-011.html
	Implementing new rigor and transparency polices in review—lessons learned
	https://www.csr.nih.gov/CSRPRP/2016/09/implementing-new-rigor-and-transparency-policies-in-review-lessons-learned/
	Updates to NIH and AHRQ RPPRs to address rigor and transparency
	https://grants.nih.gov/grants/guide/notice-files/NOT-OD-16-031.html
	FAQs on consideration of relevant biological variables, such as sex
	https://grants.nih.gov/reproducibility/faqs.htm#IV
	FAQs about SABV policy
	https://orwh.od.nih.gov/research/sex-gender/nih-policy/faq/
	https://nexus.od.nih.gov/all/tag/sabv/
	Valid analysis guidance
	https://grants.nih.gov/sites/default/files/Valid%20analysis%20CTgov%20guidance%20final_508c.pdf
	Online courses for continuing medical education credit
	https://orwh.od.nih.gov/career-development-education/e-learning
	Guidelines for researchers
	https://orwh.od.nih.gov/resources/pdf/NOT-OD-15-102_Guidance.pdf
	Guidelines for reviewers
	https://grants.nih.gov/grants/peer/guidelines_general/SABV_Decision_Tree_for_Reviewers.pdf
	RFI: Consideration of sex as a biological variable in biomedical research
	https://grants.nih.gov/grants/guide/notice-files/NOT-OD-14-128.html
	Publications
	National Research Council. (2001) *Exploring the Biological Contributions to Human Health: Does Sex Matter?* National Academies Press, Washington, DC.
	https://www.nap.edu/catalog/10028/exploring-the-biological-contributions-to-human-health-does-sex-matter
	Clayton JA. Studying both sexes: a guiding principle for biomedicine.
	http://www.fasebj.org/content/30/2/519.full.pdf+html
	Clayton JA, Collins FS. Policy: NIH to balance sex in cell and animal studies.
	http://www.nature.com/news/policy-nih-to-balance-sex-in-cell-and-animal-studies-1.15195
	Trans-NIH Strategic Plan for Women’s Health Research
	https://orwh.od.nih.gov/about/trans-nih-strategic-plan-womens-health-research
	Videos on methods and techniques
	https://orwh.od.nih.gov/research/sex-gender/methods-and-techniques/
	SABV workshop
	https://videocast.nih.gov/summary.asp?Live=26050&bhcp=1
IGH	Online interactive courses on integrating sex and gender in biomedical research, primary data collection in humans, and secondary data analysis
	http://www.cihr-irsc.gc.ca/e/49347.html
	Instructions for peer review assessment
	http://www.cihr-irsc.gc.ca/e/50837.html
	Knowledge translation
	http://www.cihr-irsc.gc.ca/e/40948.html
	SGBA action plan and resources
	http://www.cihr-irsc.gc.ca/e/50837.html
	Fact vs fiction information sheets on sex and gender
	http://www.cihr-irsc.gc.ca/e/49629.html
	Casebook: What a difference sex and gender make
	http://www.cihr-irsc.gc.ca/e/44082.html
	Publications:
	Haverfield J, Tannenbaum C. (2021) A 10-year longitudinal evaluation of science policy interventions to promote sex and gender in health research. https://doi.org/10.1186/s12961-021-00741-x
	Tannenbaum C, Ellis RP, Eyssel F, et al. (2019) Sex and gender analysis improves science and engineering.
	https://www.nature.com/articles/s41586-019-1657-6
	Tannenbaum C, van Hoof K. (2018) Effectiveness of online learning to integrate sex and gender in grant proposals.
	https://bsd.biomedcentral.com/articles/10.1186/s13293-018-0197-3
	Day S, Mason R, Tannenbaum C, Rochon PA. (2017) Essential metrics for assessing sex & gender integration in health research proposals involving human participants.
	https://journals.plos.org/plosone/article?id=10.1371/journal.pone.0182812
	Tannenbaum C, Day D; Matera Alliance. (2017) Age and sex in drug development and testing
	https://www.sciencedirect.com/science/article/pii/S104366181730275X?via%3Dihub
	Clayton JA, Tannenbaum C. (2016) Reporting sex, gender, or both in clinical research?
	https://jamanetwork.com/journals/jama/fullarticle/2577142
	Tannenbaum C, Greaves L, Graham ID. (2016) Why sex and gender matter in implementation research.
	https://bmcmedresmethodol.biomedcentral.com/articles/10.1186/s12874-016-0247-7
	Tannenbaum C, et al. (2016). Evaluating sex as a biological variable in preclinical research: the devil in the details.
	https://www.ncbi.nlm.nih.gov/pmc/articles/PMC4750169/
	Videos
	Peer-review assessment criteria
	https://www.youtube.com/watch?v=Hlceez1Dx5E&index=1&list=PL6hlpNDF2-NUIAX5AfUzs72esOD28bPQJ
	Learning about sex and gender
	http://www.cihr-irsc.gc.ca/e/50003.html
	Shaping science for a healthier world
	https://www.youtube.com/watch?v=LCiSytha55U
	GenPort—online community of practitioners for sharing knowledge and inspiring collaboration
	www.genderportal.eu
	Gender toolkit
	http://www.yellowwindow.com/genderinresearch/
	Responsible research and innovation tools
	http://www.rri-tools.eu/
	Cost Action Gender STE
	http://www.cost.eu/about_cost/strategy/targeted_networks/genderste
EC	Gendered Innovations publication, Gendered innovations: how gender analysis contributes to research (2013)
	https://ec.europa.eu/research/science-society/document_library/pdf_06/gendered_innovations.pdf
	Gendered Innovations 2 publication, Gendered Innovations 2: how inclusive analysis contributes to research and innovation (2020)
	https://ec.europa.eu/info/sites/info/files/research_and_innovation/strategy_on_research_and_innovation/documents/ki0320108enn_final.pdf
	Gendered Innovations website (2011-) with general and specific methods for sex, gender, and intersectional analysis, and case studies in various research areas
	http://genderedinnovations.stanford.edu/
	Gender-NET ERA-NET
	http://www.gender-net.eu/?lang=en
	Policy documents
	http://ec.europa.eu/research/swafs/index.cfm?pg=policy&lib=gender
	GenPort—online community of practitioners for sharing knowledge and inspiring collaboration
	www.genderportal.eu
	Gender toolkit
	http://www.yellowwindow.com/genderinresearch/
	Responsible research and innovation tools
	http://www.rri-tools.eu/
	Cost Action GenderSTE
	http://www.cost.eu/about_cost/strategy/targeted_networks/genderste

Abbreviations: AHRQ, Agency for Healthcare Research and Quality; FAQ, frequently asked questions; IGH, Institute of Gender Health; NIH, US National Institutes of Health; ORWH, Office of Research on Women’s Health; RFI, request for information; RPPR, Research Performance Progress Reports; SABV, sex as a biological variable;

During the COVID-19 pandemic, ORWH expanded the scope of its sex and gender research programs to encompass SARS-CoV-2 infection, developed guiding principles on how SABV and inclusion policies could inform COVID-19 studies to improve outcomes for everyone, and developed web content and seminars outlining the influences of sex, gender, and intersectionality on outcomes for diverse women (29).

### Canadian Institutes of Health Research Institute of Gender and Health

When the SGBA policy was introduced in 2010, applicants for health-research funding were asked to explain how their study design accounts for sex and/or gender (see Table 2). By adding that question, the number and proportion of applicants answering “yes” across all research areas nearly doubled. In the first year, the percentage of applicants who accounted for sex or gender was 20% to 50%, with the lowest proportion in basic science and higher proportions in clinical, health systems and policy research, and population health (30). Now, the percentage exceeds 75% in basic sciences and 80% to 90% in the clinical sector and population health. Since 2000, the IGH has invested $8 million per year from the CIHR’s priority-driven budget to promote SGBA in research. Incentives include funding opportunities with SGBA as the primary objective, such as catalyst grants for basic scientists who have not yet factored sex into their experiments or who require funds to include female cells or animals. Team grants explore the added value of accounting for gender in human health interventions. SGBA requirements are embedded at various points within the request for applications, as a principle of funding, and in evaluation criteria. IGH funding investments are leveraged by other institutes and external partners. The IGH also holds workshops for new investigators to coach them on integrating sex and gender into their research programs.

The IGH launched 2 capacity-building mechanisms to augment researchers’ ability to integrate and critically appraise sex and gender in research studies (31). In 2014, the IGH began requiring the inclusion of a sex and gender champion on the research team, platform, or consortium for select large grant competitions. The champion ensures that sex and/or gender considerations are integrated into every step of the research project, including project rationale, experimental design, methods, analysis, and knowledge translation and dissemination (32). Champions also act as coaches, set best practices for sex and gender science, and participate as members of a community of practice.

To increase the capacity of peer reviewers to assess the quality of integration of sex and gender analyses, the CIHR’s SGBA Research Action Plan changed the peer reviewer assessment form for investigator-initiated project competitions to require peer reviewers to rate the integration of sex and gender as a strength, weakness, or not applicable to the application, and to recommend improvements. The plan also requires peer-review committee chairs and scientific officers for the project competitions to complete one of the IGH’s online training modules, and for each committee to undergo an in-person coaching session. A sex-and-gender expert is also assigned to each peer-review committee to ensure the discussions around the appropriate inclusion or omission of sex and gender meet the highest scientific standards. CIHR applicants for specific grant competitions must provide proof of completion of at least one online training module to be eligible to submit an application. The modules effectively increased researcher and peer-reviewer knowledge and skills around sex and gender integration and assessment (33). In its new strategic plan for 2021 to 2031, the CIHR amplifies the focus on equity in research with a commitment to further refine concepts of research excellence, improve the diversity of research teams, and codify consideration of sex and gender in research (19).

During the COVID-19 pandemic, the CIHR implemented new gender policies to support female scientists and to improve the integration of sex and gender in COVID-19–related research (18). The changes included extending the application time window, reducing administrative burden, doubling parental leave credits, providing supplements for factoring in biological sex, providing specific guidance to applicants and reviewers on how to integrate sex and gender in COVID -19 research (34), and cracking down on evaluation criteria. Following these changes, the proportion of female applicants increased by 29% to 39%, and the proportion of successful applications led by a female investigator doubled from 22% to 45%. Consideration of biological sex increased from 55% to 94% in funded applications, and applications indicating that sex was considered in the proposed work were more likely to be funded (odds ratio 3.13, 95% CI, 1.57-6.23).

### European Union European Commission

The EC relaunched its “gender dimension in research” policy with Horizon 2020 (2014-2020; see Table 1). Requirements and guidance for integrating sex and/or gender analysis into research were summarized in a vademecum that describes the integration process for each stage of the research cycle—from programming through implementation, monitoring, and evaluation (35). It became apparent that the research community required methodological tools for integrating SGBA into basic and applied research. To address these needs, the EC convened an expert group entitled Innovation through Gender that joined the Gendered Innovations project to produce a peer-reviewed website (36) and a report, “Gendered Innovations: How Gender Analysis Contributes to Research” (37). More than 60 basic scientists, engineers, and gender experts developed methods of sex and gender analysis and contributed to a set of case studies that demonstrate how sex and gender analyses add value to research and innovation in terms of excellence, creativity, societal relevance, and business opportunities. Additional tools and guidance to assist researchers preparing proposals for Horizon 2020 were developed by, among others, the GENDER-NET ERA-NET project (38).

In the Horizon 2020 work programmes, topics with an explicit gender dimension were “flagged” as fields where research could benefit from integrating SGBA. These fields included computing, nanotechnology, oceanography, geosciences, organic chemistry, aeronautics, space medicine, biodiversity, ecology, and biophysics, among others. For gender-flagged topics, applicants were required to integrate sex and gender analysis into their research design. In 2018, the proportion of flagged topics, by field of study, was 8% of energy topics, 34% of climate topics, 60% of health topics, and 78% of social science studies.

To support implementation, Horizon 2020 funded the Advisory Group on Gender, consisting of gender experts from each research domain. This group produced position papers that suggested ways to better integrate the gender dimension into each research domain and societal challenge (39). The advisory group also addressed the issue of gender bias in proposal evaluations (40) and advised EC staff on how to develop briefings for evaluators on the gender dimension relevant to their particular domain.

In the EU, an interim evaluation of the gender dimension as a cross-cutting issue revealed that the proportion of gender-flagged topics in the work programmes increased from 16% (99 of 610 topics) in 2014 to 2015, to 19% (108 of 568 topics) in 2016 to 2017, and to 23% (110 of 473 topics) in 2018 to 2019. Among individual projects, 32.4% were identified by project officers as including a gender dimension. Further, a qualitative analysis of a subset of 111 projects from gender-flagged topics showed that 53% included the gender dimension either wholly or in part (41). Under Horizon 2020, research applications could include training on the gender dimension as an eligible cost. Since none of the 111 projects mentioned here included gender training, more action is needed to make this funding better known. To address that, an expert group developed metrics to grade proposals on how well the gender dimension is incorporated into the whole research process (42).

The EC just released its new funding framework: Horizon Europe (2021-2027), the largest EC research and innovation programme to date, with a budget of nearly €95.5 billion. To further the gender dimension, the EC convened an expert group again in 2018 to 2020 and published a report, “Gendered Innovations 2: How Inclusive Analysis Contributes to Research and Innovation” (43). Although the policy is not finalized, it will likely require all proposals (not simply flagged topics) to include sex and gender analysis or to explain why such analysis is not relevant. This is a major step forward, but more is needed. To create a multiplier effect, the EU established a new eligibility requirement: Applicants who wish to apply for Horizon Europe funding must implement a gender equality plan. This new requirement will be put in place gradually, by first addressing public bodies, research organizations, and institutions of higher education, with a transition period of 1 year before it is fully enforced.

## Progress Made, Lessons Learned, and Next Steps

Much progress has been made in terms of integrating sex and gender considerations into research. Awareness of this issue within the scientific community has increased by requiring the inclusion of both sexes in clinical and preclinical studies. In Table 2, we highlight individual policies, mechanisms, and lessons learned by each agency that could be considered by other entities when trying to promote the integration of sex and gender into research. Our agencies implemented policies and requirements; provided incentives, resources (Table 3), and guidance; performed outreach; and developed training.

To ensure accountability, it is essential to train evaluators to critically appraise the appropriate integration of sex and gender in proposals. Once applicants realize that high-quality integration is associated both with improved research quality and funding success, as occurred with the CIHR’s new COVID-19 research policies and the NIH’s SABV policy, the practice will be institutionalized. A key lesson that each agency learned was that such a multipronged approach using both incentives and requirements is needed to change minds, attitudes, behaviors, and research strategies across the scientific community. In addition, each country has its unique culture and limits, which led to certain policy adjustments to fit the context of those barriers or opportunities. A universal lesson—among researchers least likely to change—was that money talks. To successfully identify which carrots and/or sticks are most effective and the context in which they work, it will be crucial to ensure uptake of, accountability for, and critical appraisal of policies and standards that integrate sex and gender considerations across the research continuum.

Globally, the Gendered Innovations project, launched at Stanford University in 2009, works with researchers and funders to develop methods of sex and gender analysis for the natural sciences, medicine, engineering, and environment (36). Gendered Innovations collaborated with the EC in 2011 to provide the intellectual foundations for the Horizon 2020 policy and again in 2018 to develop new materials for Horizon Europe (43). Gendered Innovations also recently partnered with the Wellcome Trust to evaluate funding agencies globally on the effectiveness of their policies for sex, gender, and diversity analysis, in efforts to share best practices. In the Netherlands, the Ministry of Health, Wellbeing and Sports funded a Gender & Health programme (2016-2020) with a budget of €12 million aimed at sex and gender analysis in research. In Asia, South Korea founded a Centre for Gendered Innovations in Science and Technology Research and is currently implementing policies for sex and gender analysis across its research funding agencies (44). The Science Council of Japan and the Japanese Science and Technology Agency recently held meetings on sex and gender analysis in research and innovation. For a full list of funding agency policies on sex and gender analysis, see reference (26).

Another lesson learned by these agencies is that they cannot go at this alone. Next steps require funding agencies to collaborate with other stakeholders, including peer-reviewed journals, researchers, universities, biomedical companies, pharmaceutical companies, institutional review boards, philanthropic groups and professional societies to adopt similar standards for considering sex and gender (1). Editors of peer-reviewed journals should ensure scientific excellence when selecting papers for publication. To this end, the CIHR, NIH, and EC work with organizations, such as the European Association of Science Editors, to encourage journals to adopt standards, such as the Sex and Gender Equity in Research guidelines (45). It is important that granting agencies and journals send a consistent message that publishing data disaggregated by sex/gender is necessary to ensure scientific excellence. Yet, journal guidelines are not always followed (46, 47).

Funding agencies must also continue to support universities’ efforts to integrate these tools into education at undergraduate, graduate, and interprofessional levels. To train the next generation, it is crucial to integrate knowledge of sex and gender analysis into core medical, natural science, and engineering courses. Yet, at present, even most medical schools do not include SABV and SGBA in their curriculum. To encourage the consideration of sex by animal care committees and sex and gender by human research ethics boards, work is ongoing with the Secretariat on Responsible Conduct of Research (in Canada).

Globally, gross domestic expenditures on research and development (R&D) are driven mainly by business and higher education, with government expenditures on R&D since 2016 starkly below spending based on sector (48). Currently, few pharmaceutical companies integrate SGBA into their research (49). In addition, globally there is underinvestment in companies and technologies relating to women’s health and in R&D efforts regarding sex differences, such as sex and gender-aware clinical trials (50). We suggest that government agencies partner with industry to share lessons learned, collaborate, and promote systemwide solutions for integrating sex and gender considerations into research and clinical studies.

Last, government agencies need to engage with professional societies, such as the Endocrine Society, to ensure lasting cultural shifts in the scientific community with regard to considering sex and gender. Recently, the Endocrine Society released a scientific statement on sex differences in research that calls for females and males to be studied thoroughly to improve public health (2). The statement assists clinicians and researchers by providing guidance and recommendations for areas of practice.

Furthermore, it is necessary to develop new quantitative tools to measure gender. One such tool was recently published (11). There is also a recognized need for methods to understand the interactions of sex and gender (2, 11, 51) and the intersections of sex and/or gender with other factors that can influence research results, such as ethnicity/race, age, socioeconomic status, geographic location, and sexual orientation, along with the intersectionality of all these variables.

Herein, we have sounded a call to action by researchers and key stakeholders globally: Government funding agencies, including the NIH, CIHR, and the EC, need your help to actualize the promise of sex and gender integration into biomedical research (52). Our agencies hope not only that others can learn from the policies and mechanisms that have been implemented but also that together we can promote systemwide solutions that integrate sex and gender considerations throughout the biomedical research enterprise. The ultimate goal is to create rigorous, responsible, and generalizable science worldwide for all.

## Data Availability

Data are available from the corresponding author. Data sharing is not applicable to this article as no datasets were generated or analyzed during the current study.

## Abbreviations

CIHR: Canadian Institutes of Health Research
EC: European Commission
EU: European Union
FDA: US Food and Drug Administration
FP: framework programme
GBA: gender-based analysis
IGH: Institute of Gender and Health
NIH: US National Institutes of Health
ORWH: Office of Research on Women’s Health
R&D: research and development
SABV: sex as a biological variable
SCOR: Specialized Centers of Research
SCORE: Specialized Centers of Research Excellence
SGBA: sex- and gender-based analysis

## Additional Information

***Disclosures:*** J.W. is employed by the NIH. C.T. is employed by the CIHR. I.K. is retired from Maastricht University, the Netherlands. L.S. is employed by Stanford University. J.C. is employed by the NIH.
